# Focal aware somatosensory seizures with paresis as a complication of surgery for chronic subdural hematoma

**DOI:** 10.1016/j.ebr.2023.100621

**Published:** 2023-09-24

**Authors:** Shohei Ishida, Naoki Nitta, Kazumichi Yoshida

**Affiliations:** a Department of Neurosurgery, Shiga University of Medical Science, Shiga, Japan; b Department of Neurosurgery, Kohka Public Hospital, Shiga, Japan

**Keywords:** Ictal paresis, Akinetic seizure, Hemiparetic seizure, Inhibitory motor seizure, Negative motor area

## Abstract

There has been only one previous published report of focal aware somatosensory seizures with paresis as a postoperative complication of chronic subdural hematoma (cSDH). This is the second case report of this condition captured on electroencephalography (EEG) as a postoperative complication of cSDH. A 76-year-old man with no history of epilepsy was taken to the emergency department of Shiga University of Medical Science Hospital because of transient weakness of the lower extremities. Head computed tomography showed bilateral cSDH that was larger on the left. Seven days after burr-hole evacuation of the left cSDH, the patient experienced a brief clonic seizure of the right hand without postoperative recurrence of cSDH. He then experienced a tingling sensation in, followed by clumsiness and weakness of, the right upper extremity without fluctuations in consciousness or convulsive movements. These symptoms appeared repeatedly, with intermittent improvement, persisting for 6 days after onset. Scalp EEG showed an electrographic seizure in the left central area, suggesting that the symptoms corresponded to focal aware somatosensory seizures with paresis. The symptoms and epileptiform patterns and electrographic seizures on the EEG disappeared with antiseizure medications.

## Highlights

• We report focal aware somatosensory seizures with paresis after surgery for cSDH.
• Seizures occurred without any imaging evidence of postoperative complications.
• The semiology was a persistent tingling sensation, local clumsiness, and weakness.
• An EEG showed a seizure pattern in the left central area.
• The symptoms and epileptiform discharges disappeared upon antiseizure treatment.

## Introduction

Early postoperative seizures have been reported as a potential complication of treatment of chronic subdural hematoma (cSDH), with a reported incidence of 0.67 %–13.6 % [1], [2]. Despite the high prevalence of cSDH ranging from 8 to 58 per 100,000 in those aged > 65 years, few previous reports have provided either detailed semiology of the postoperative seizures or detailed findings of electroencephalography (EEG) in patients treated surgically for cSDH [3], [4].

Focal aware somatosensory seizures with paresis, formerly called focal negative motor (akinetic) seizures, are rare and are probably caused by abnormal activation of the somatosensory area and the negative motor area [4], [5]. This is the second published case report in which focal aware somatosensory seizures with paresis after surgery for cSDH have been captured on EEG, the first such report having been published in 2006 [4].

## Case report

**Fig. 1:**
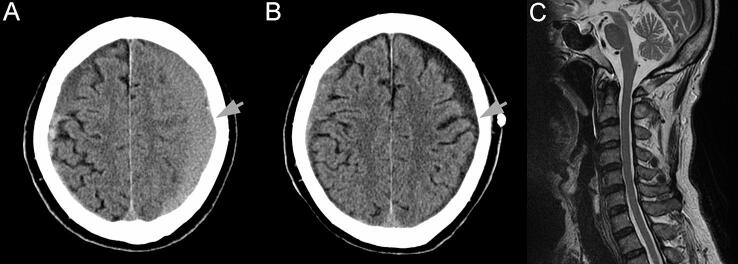
Head computed tomography (CT) and cervical spine magnetic resonance imaging (MRI). (A, B) Axial CT of the head before (A) and 7 days after (B) surgery. Note the absence of recurrence of the subdural hematoma and the absence of any apparent lesions in the brain on the left side (arrows). (C) Sagittal MRI of the cervical spine 3 days after symptom onset. Neither spinal cord compression nor intra-axial lesions were observed.

A 76-year-old man was brought to the Emergency Department of Shiga University of Medical Science Hospital because of transient weakness of the lower extremities and gait disturbance. The patient had past histories of diabetes and hyperlipidemia, but not epilepsy. Computed tomography (CT) of the head showed bilateral cSDH, larger on the left side (Fig. 1A). The patient was admitted and the left hematoma was surgically drained without any complication or postsurgical deficit.

The patient had had no postsurgical deficits until 7 days after the procedure, when he experienced a brief episode of a clonic seizure of the right hand without any postoperative recurrence of cSDH on CT (Fig. 1B). After the clonic seizure stopped, the patient experienced a persistent tingling sensation and pain in the right upper extremity and shoulder, as well as clumsiness and weakness of the right upper extremity, without any fluctuation in consciousness. Postictal Todd’s paralysis was suspected, but the patient experienced repeated improvement (sometimes disappearance) and exacerbation of the symptoms for 6 days after onset. Furthermore, the patient experienced both negative motor symptoms (clumsiness and weakness) and positive sensory symptoms (tingling sensation and pain), which is atypical for Todd’s paralysis.

On examination of the patient 2 days after the onset of symptoms, the tingling sensation and pain were localized in the right upper extremity and shoulder. Hypoesthesia was also observed on the radial side of the right hand. The patient could move his right hand voluntarily, but the movement was slow, weak, and awkward. The grip strength of the right hand, which had been 24 kg before the onset of the symptoms, had decreased to 10 kg. Tendon reflexes were normal and no pathological reflexes were observed. The symptoms were stereotypical, occurred in the same parts of the body, and cycled through improvement and exacerbation. A CT of the head was performed again, but, as previously, it showed no recurrence of cSDH or acute brain lesions.

**Fig. 2:**
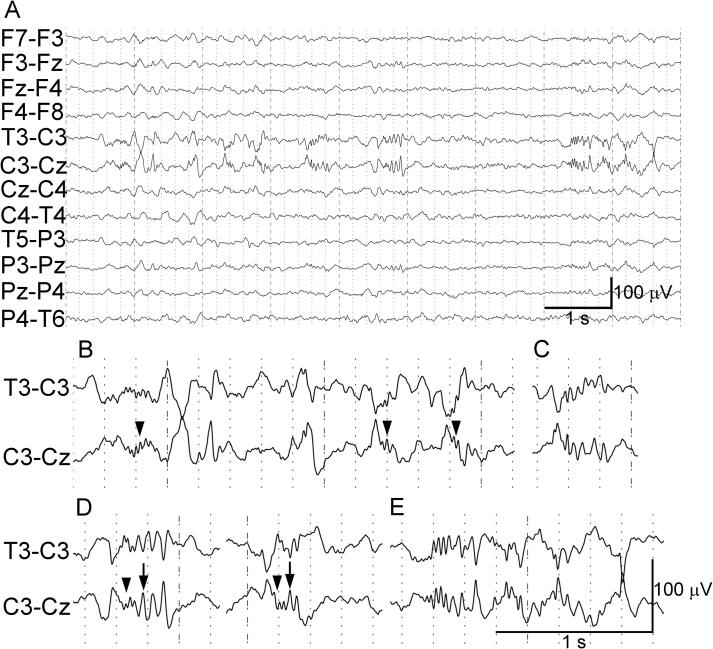
Epileptiform pattern shown on a transverse bipolar montage 3 days after symptom onset. (A) Epileptiform patterns immediately before an electrographic seizure starts. Phase reversals are seen at C3. (B–E) Each epileptiform discharge. (B) Delta or theta waves superimposed with low-voltage fast activities (arrowheads) of approximately 25–30 Hz, lasting ≤ 0.4 s, which are sometimes followed by slow waves. Sharp waves are also observed. (C) A 15- to 25-Hz polyspike-and-slow-wave complex. (D) Low-voltage fast activities (arrowheads) followed by a polyspike complex (arrows). (E) Brief potentially ictal rhythmic discharges.

**Fig. 3:**
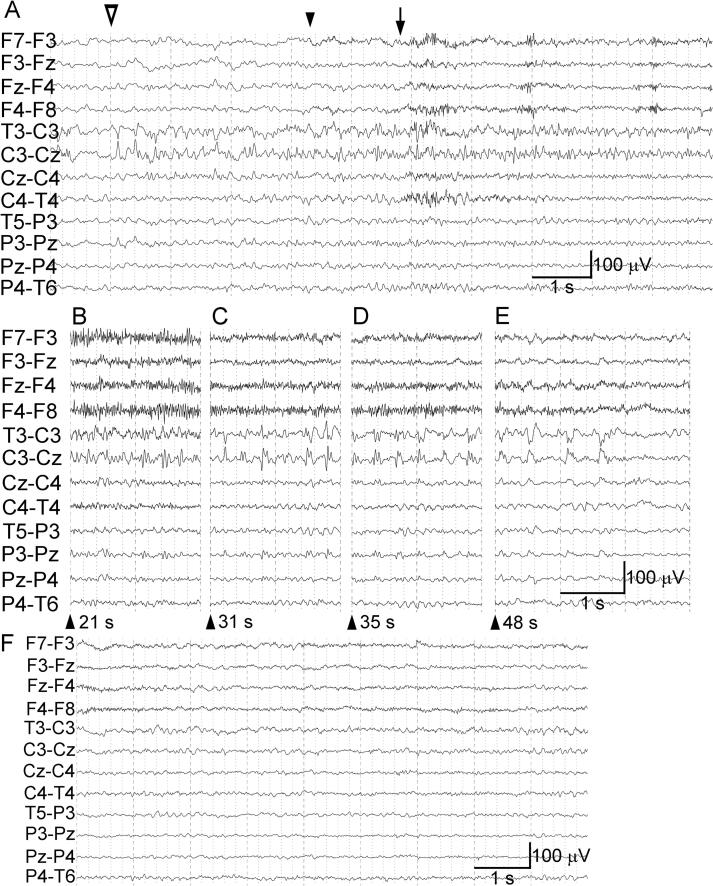
Electrographic seizure shown on a transverse bipolar montage 3 days after symptom onset. (A) Continuation of Fig. 2A, showing the start of an electrographic seizure (open arrowhead). Quasi-rhythmic spikes and theta waves of approximately 6 Hz appear in the left centroparietal region. Phase reversals are seen at C3. Quasi-rhythmic spikes increase in frequency (∼8 Hz) and decrease in amplitude, at which time the right hand of the patient felt strange and he rubbed his right thumb and index finger (at the arrowhead). The quasi-rhythmic spikes further increase in frequency (∼15 Hz) and decrease in amplitude, and the patient slightly moved his mouth and arms because of the start of a tingling sensation, pain, and stiffness in the right upper extremity, which resulted in interference from muscle activity on the EEG (arrow). The quasi-rhythmic spikes fluctuate in frequency and amplitude in the latter part of the trace. (B) The quasi-rhythmic spikes are interposed with larger spikes or polyspike complexes. (C) The lower-amplitude quasi-rhythmic spikes fade out, with the interposed spikes or polyspike complexes appearing more clearly. (D) The ictal discharges change into 3-Hz quasi-periodic polyspike complexes. (E) The quasi-periodic polyspike complexes exhibit a gradual decrease in frequency (from 3 to 2 Hz) and change in morphology, and they stop 50 s after the onset of ictal activity. (F) Continuation of (E). After the end of the electrographic seizure, the background activity is almost symmetric in voltage, and no breach rhythm is observed. Each arrowhead in (B–E) indicates the time after the onset of ictal activity.

Three days after symptom onset, the frequency and duration of the symptoms were reduced, but the symptoms were occurring approximately once or twice per hour. Another differential diagnosis besides Todd’s paralysis was cervical spondylotic radiculopathy, but magnetic resonance imaging of the neck on the same day showed no cervical lesion (Fig. 1C). Nonepileptic transient neurological symptoms (TNS) or acute symptomatic seizures were also suspected, and routine scalp video-EEG was performed on the same day. The C3 electrode was placed away from the cSDH burr hole and wound. The video-EEG showed occasional epileptiform patterns (Fig. 2A), as well as an electrographic seizure (Fig. 3A–E) [6], [7]. The patient experienced an episode of tingling sensation, pain, and stiffness in the right upper extremity simultaneous with the electrographic seizure; this was confirmed retrospectively in the video. Epileptiform patterns were observed occasionally, peaking at the C3 electrode; low-voltage fast activities and sharp waves (Fig. 2B), polyspikes (Fig. 2C, D), and brief potentially ictal rhythmic discharges (Fig. 2E) [6], [7] were found.

An electrographic seizure was recorded as follows: quasi-rhythmic spikes and theta waves at approximately 6 Hz appeared in the left centroparietal region and were maximal at the C3 electrode (Fig. 3A, open arrowhead; Fig. 3A is a continuation of Fig. 2A). The frequency of the quasi-rhythmic spikes increased (∼8 Hz) and their amplitude decreased (Fig. 3A, arrowhead), at which time the right hand of the patient felt strange and he rubbed his right thumb and index finger, as recorded in the video and retrospectively reported by the patient. The frequency of the quasi-rhythmic spikes then increased further (∼15 Hz) and their amplitude decreased, at which time the patient moved his mouth and arms slightly because of the start of the tingling sensation, pain, and stiffness in the right upper extremity, with the muscle activity interfering with the EEG (Fig. 3A, arrow). The frequency and amplitude of the quasi-rhythmic spikes then fluctuated (Fig. 3A, latter part), and these spikes were interposed with larger spikes or polyspike complexes (Fig. 3B). The lower-amplitude quasi-rhythmic spikes faded out, with the interposed spikes or polyspike complexes appearing more clearly (Fig. 3C), and the pattern then changed into 3-Hz quasi-periodic polyspike complexes (Fig. 3D). The quasi-periodic polyspike complexes showed a gradual decrease in frequency (from 3 to 2 Hz) and change in morphology, and they stopped 50 s after the onset of ictal activity (Fig. 3E). The symptoms improved after the end of the electrographic seizure. In addition to the epileptiform patterns or the electrographic seizure, single theta waves were frequently observed at C3, but the background activity was almost symmetric in voltage, and no breach rhythm was observed (Fig. 3F). The EEG showed only one electrographic seizure, but the symptoms were the same as the previous stereotypical symptoms, so we concluded that electrographic seizures like this had been occurring repeatedly, resulting in the recurring symptoms. Because the patient had experienced a somatosensory aura and ictal paresis during the recorded seizure, his symptoms were diagnosed as corresponding to focal aware somatosensory seizure with paresis, also known as “ictal paralysis” [8] or “akinetic seizure” [9].

Three days after symptom onset, the patient was started on antiseizure medication (lacosamide 100 mg/day—the maximum starting dose allowed in Japan), but the symptom frequency and duration had not declined by the following day. In Japan, lacosamide doses cannot be increased daily (with the permitted increase being 100 mg/week), so levetiracetam (1000 mg/day; the maximum starting dose allowed in Japan) was added. Seven days after symptom onset (4 days after the start of antiseizure medications), all the symptoms of the right upper extremity disappeared, and a scalp EEG showed neither epileptiform patterns nor electrographic seizures; since then, the patient has not experienced any of the abovementioned symptoms over a period of more than 2 years. Four days after the start of antiseizure medications, the lacosamide was stopped and the levetiracetam dose was reduced to 750 mg/day because of the exacerbation of dizziness and gait disturbance. The dose of levetiracetam was gradually tapered over a period of 6 months, after which it was stopped without any recurrence of seizures afterwards.

## Discussion

This is the second published case report of focal aware somatosensory seizures with paresis as a postoperative complication in cSDH; they were first reported by Villani et al., [4].

In this case, the patient experienced a tingling sensation and motor weakness in the right upper extremity after an initial clonic seizure 7 days after surgery for left cSDH. The symptoms repeatedly abated then returned, and epileptiform patterns and an electrographic seizure were recorded on an EEG. Four days after the start of antiseizure medications, all symptoms disappeared, with normalization of the EEG.

Temporary neurological symptoms—especially negative symptoms—have been described as cerebral transient ischemic attacks (TIAs), transient neurological deficits, and TNS [10]. TNS have been reported to occur in 8.6 % of preoperative and 5 % of postoperative cSDH patients [10], [11]. Nonepileptic TNS or TIAs in preoperative cSDH patients have been cured after evacuation of the subdural hematoma [12], [13], [14], whereas nonepileptic TNS or TIAs in postoperative cSDH patients have often been attributed to seizures despite an atypical semiology or evolution, and they have not responded to antiseizure medications [15]. Patients with SDH-associated nonepileptic TNS are likely to have negative symptoms, prolonged symptom duration (≥5 min), and dysphasia, whereas patients with SDH-associated seizures are likely to have positive symptoms, clonic movement, and impaired awareness [16]. EEGs in cSDH patients with nonepileptic TNS or TIA show negative findings, such as lateralized slowing [11], [12], [16], [17]. Recent studies suggest that a substantial proportion of cSDH-related TNS represents the clinical manifestation of underlying cortical spreading depolarization [11], [15]. Although the negative motor symptoms and prolonged symptomatology in our patient were atypical of seizure activity [16], the EEG showed epileptiform patterns and an electrographic seizure. Furthermore, the patient’s symptoms were time-locked with the electrographic seizure, suggesting that electrographic seizures such as this one had caused the continued stereotypical symptoms from the onset. We speculate that, during the first 2 days after symptom onset, the patient’s seizures were so frequent that the symptoms cycled through improvement and exacerbation.

Gowers first described seizures characterized by paresthesia and motor weakness of a limb without convulsions [18]. Subsequently, such rare seizures have been reported as “ictal paralysis” [8], “akinetic seizure” [9], and, here, as focal aware somatosensory seizures with paresis. Akinetic seizure is defined by the inability of individuals to perform voluntary movements, but not because of a loss of consciousness or the loss of muscle tone [5], [19]. The symptomatogenic zone for akinetic seizures has been reported to be negative motor areas [5], although Penfield and Jasper “found no evidence that an inhibitory portion is separable, as a distinct area, from the activating portion” [8]. Although the location of negative motor areas may differ among individuals, these areas have been found in the premotor area, precentral gyrus, postcentral gyrus, pre-supplementary motor area, and posterior parietal cortices [9], [20], [21], [22]. A maximal seizure pattern in the central area in our patient is consistent with these hypothetical symptomatogenic zones in the postcentral gyrus, precentral gyrus, or premotor area.

Somatosensory symptoms concomitant with ictal paresis in the same body part, as experienced by our patient, have been described previously [8], [18]. These somatosensory symptoms were probably caused by abnormal activation of the primary somatosensory area, as shown by the maximal seizure pattern in the central area in our patient, and they were equivalent to somatosensory seizures. Somatosensory seizures consist of abnormal sensations, with the most common epileptic somatosensory perception being paresthesia (“tingling”) [5]. In our patient, there were symptomatogenic zones, probably in the primary somatosensory area (postcentral gyrus) and the negative motor area, as described above. The postcentral gyrus, precentral gyrus, and premotor area have functionally and structurally reciprocal connections with each other [23], suggesting that, in our patient, the somatosensory area of the right upper extremity and the negative motor area of the same body part may be closely connected and simultaneously abnormally activated via those connections.

In patients with cSDH, perioperative seizures in the form of generalized seizures, focal motor seizures, or focal somatosensory seizures have been reported [24]. However, focal aware somatosensory seizure with paresis is a rare seizure type, and only one previous report has described a similar case of focal aware somatosensory seizures with paresis related to the postoperative course of cSDH [4]. In that report, the patient continued to experience brief episodes of sudden tingling and numbness in his left arm, starting in the hand, followed by prolonged severe flaccid paresis associated with a complete loss of superficial and deep sensation without loss of consciousness [4]. The EEG showed low-voltage fast activity on the right centrofrontal leads coinciding with the tingling in the left arm, as well as an ictal discharge of repetitive spikes involving the right frontal, central, and parietal regions that was accompanied by the motor deficit [4]. The severity of the symptoms and the spreading of the ictal discharges on the EEG differed between our patient and that in the report of Villani et al., [4], but the symptoms were otherwise similar to those reported previously and a phase reversal on the EEG was observed in the central area in both cases, suggesting that the discharges originated in the perirolandic cortices involved in the sensorimotor system, and that differences in the strength or spread (or both) of the paroxysmal discharges may have affected the severity of the symptoms.

Seizures after surgery for cSDH usually occur only once [25], or fewer than five times [16]. Therefore, a patient’s epileptic discharges may have already disappeared by the time an EEG was performed, and for this reason neither the detailed semiology nor the EEG findings have been sufficiently reported. As described above, transient negative motor symptoms are often diagnosed as TIA, and a timely EEG is often not performed. Furthermore, seizures seldom occur during routine scalp EEGs. There may therefore be more cases of focal aware somatosensory seizures with paresis after cSDH surgery, but these cases may resolve before the seizures are captured on an EEG. Our patient’s symptoms were not clinically typical of seizures, and they continued over a period of 6 days. Seizures were not suspected at first, but the continuous nature of the symptoms enabled us to perform an EEG before the symptoms disappeared. Fortunately, an electrographic seizure with symptoms was captured in the first video-EEG examination, enabling a diagnosis to be made. The symptoms and epileptiform patterns and electrographic seizures on the EEG disappeared completely with antiseizure medications, thus making the diagnosis final.

In our patient the quasi-rhythmic spikes looked like a breach rhythm. However, the C3 electrode was placed away from the burr hole. On the EEG, the background activity was almost symmetric in voltage at the C3 and C4 electrodes when epileptiform discharges were absent. Furthermore, the ictal activity was accompanied by simultaneous stereotypical symptoms. Together, these observations indicated that the quasi-rhythmic spikes were not a breach rhythm, but an electrographic seizure. This seizure already fulfilled both criteria for the definition of electrographic seizure, namely the presence of epileptiform discharges averaging > 2.5 Hz for ≥ 10 s and the presence of sequential changes in frequency and morphology (Fig. 3A–E) [7].

This case report does have some limitations. First, there was no long-term video-EEG monitoring, which may have revealed the relationship between the symptoms and electrographic seizures on the EEG more precisely. However, because the symptoms persisted for 6 days, an electrographic seizure could be detected on routine scalp EEG, and the disappearance of this seizure concurrently with the disappearance of symptoms was confirmed. Second, the moment of cessation of seizures and the simultaneous cessation of the electrographic seizure on the EEG was not confirmed by intravenous administration of benzodiazepine. As the patient was elderly and unsteady, intravenous benzodiazepine was not administered because it was believed, although tentatively, that the benzodiazepine could have aggravated the unsteadiness and resulted in the patient becoming bedridden.

## Conclusion

Focal aware somatosensory seizures with paresis are rare. This is the second published report of this condition as a postoperative complication of cSDH. EEG data suggested that paroxysmal activation in the perirolandic cortices may have caused this semiology.

## Ethical statement

Written informed consent to publish this information was obtained from the patient.

## Funding

This research did not receive any specific grants from funding agencies in the public, commercial, or not-for-profit sectors.

## CRediT authorship contribution statement

**Shohei Ishida:** Data curation, Writing – original draft. **Naoki Nitta:** Resources, Investigation, Data curation, Supervision. **Kazumichi Yoshida:** Writing – review & editing.

## Declaration of Competing Interest

The authors declare that they have no known competing financial interests or personal relationships that could have appeared to influence the work reported in this paper.
